# CT-Quantified Sarcopenic Visceral Obesity Is Negatively Associated with Recompensation in Patients with Decompensated Cirrhosis: A Retrospective Single-Center Study

**DOI:** 10.3390/jcm15124482

**Published:** 2026-06-10

**Authors:** Hongxia Zhang, Zhenzhen Wen, Fengjuan Tian, Yanfei Fang

**Affiliations:** 1 Department of Gastroenterology, Sir Run Run Shaw Hospital, Zhejiang University School of Medicine, Hangzhou 310058, China; zhanghongxia0926@zju.edu.cn (H.Z.); zhenzhenwen@zju.edu.cn (Z.W.); 2 Department of Radiology, Sir Run Run Shaw Hospital, Zhejiang University School of Medicine, Hangzhou 310058, China

**Keywords:** decompensated cirrhosis, recompensation, sarcopenia, visceral obesity

## Abstract

**Background/Objectives**: Recompensation in patients with decompensated cirrhosis has significant prognostic implications. In this study, we aimed to evaluate the incidence and predictors of recompensation in cirrhotic patients, specifically focusing on elucidating the influence of sarcopenia and visceral obesity on achieving recompensation in a cohort of decompensated individuals. **Methods**: We conducted a retrospective analysis of 195 patients with decompensated cirrhosis from 2021 to 2024. Body composition abnormalities were determined by the skeletal muscle index (SMI) and visceral-to-subcutaneous adipose tissue ratio (VSR) on computed tomography (CT), respectively. Factors related to recompensation, defined using the modified Baveno VII criteria, were identified using multivariate regression. **Results**: Patients who achieved recompensation exhibited a lower age (62 vs. 67, *p* < 0.05), a higher body mass index (22.8 vs. 21, *p* < 0.01), a lower aspartate aminotransferase level (32 vs. 39, *p* < 0.01), a higher albumin level (35.2 vs. 32.3, *p* < 0.01), a lower ascites prevalence (60% vs. 74.07%, *p* < 0.05), a lower Child–Pugh score (6 vs. 7, *p* < 0.01), and a lower End-Stage Liver Disease score (9 vs. 10, *p* < 0.05) compared to those with non-recompensated cirrhosis. Body composition abnormalities were significantly more prevalent in non-recompensated patients than in recompensated patients (77.04% vs. 63.33%, *p* < 0.05), mainly because of a significantly higher prevalence of combined sarcopenia and visceral obesity in non-recompensated individuals (28.29% vs. 6.67%, *p* < 0.01). Multivariate analysis indicated that combined sarcopenia and visceral obesity was the sole independent risk factor for non-recompensation in this population. Furthermore, in the subgroup of patients aged < 70 years, with normal weight and preserved liver function, differences in recompensation rates among various states of body composition abnormalities were more pronounced. **Conclusions**: Sarcopenic visceral obesity is an independent risk factor for non-recompensation in patients with decompensated cirrhosis, highlighting the need for targeted interventions to mitigate body composition abnormalities in this vulnerable population.

## 1. Introduction

Cirrhosis is the common terminal stage of various chronic liver diseases. Clinically, cirrhosis is typically divided into two stages: compensated and decompensated [1]. Traditional views hold that once cirrhosis enters the decompensated phase, liver dysfunction is often irreversible. However, an increasing number of studies show that some patients with decompensated cirrhosis can achieve improvement in liver function and relief of clinical symptoms, even recover to a clinical state approaching compensation, after effective control or elimination of the underlying cause and active supportive treatment [2,3,4]. As a result, the concept of “recompensation” has gradually emerged and attracted widespread attention [5]. However, a subset of patients fails to attain recompensation despite successful control of the etiology and management of complications, suggesting the existence of unexplored factors for recompensation in cirrhotic patients.

Body composition refers to the content, proportion, and distribution of bone, muscle, and fat tissue in the human body. The association between abnormal body composition and poor prognosis in patients has been widely confirmed [6,7,8]. Recently, the synergistic effect of sarcopenia coexisting with obesity has become a research hotspot. Some studies suggest that the synergistic effect of these two conditions is greater than the individual effects of sarcopenia or obesity alone [9,10,11]. On one hand, visceral fat accumulation releases various adipokines and simultaneously leads to infiltration of pro-inflammatory macrophages and other immune cells, thereby creating a chronic low-grade inflammatory environment [12]. On the other hand, fat tissue may accumulate within skeletal muscle, secrete and release certain pro-inflammatory cytokines, ultimately leading to skeletal muscle dysfunction [13]. These pro-inflammatory cytokines, in turn, exacerbate adipose tissue inflammation, resulting in a vicious cycle that contributes to adverse prognosis.

While several studies have investigated the role of body composition abnormalities in the progression of liver cirrhosis, the combined effects of sarcopenia and visceral obesity on recompensation in cirrhosis remain unexplored [8,14,15,16,17]. This study aimed to evaluate the incidence and predictors of recompensation in decompensated cirrhosis, following the Baveno VII definition of recompensation. It specifically focused on elucidating the influence of three distinct body composition abnormalities, including isolated sarcopenia (I-sarco), isolated visceral obesity (I-VO), and their coexistence, on achieving recompensation in cirrhotic patients. Characterizing these body composition abnormalities could reveal novel therapeutic targets to improve prognosis in this vulnerable cohort.

## 2. Materials and Methods

### 2.1. Participants and Evaluations

This retrospective analysis enrolled patients diagnosed with decompensated cirrhosis from 2021 to 2024, hospitalized for symptoms including ascites, esophagogastric variceal bleeding (EGVB), hepatic encephalopathy (HE) and infections. All included patients presented with splenomegaly and portal hypertension on CT imaging. Exclusion criteria were: (1) hepatocellular carcinoma or other malignancies; (2) acute-on-chronic liver failure; (3) concomitant neuromuscular disease; (4) a history of serious extrahepatic diseases; (5) lack of comprehensive data from computed tomography (CT) scans; (6) undergoing liver transplantation; (7) death before achieving recompensation. This study received written ethics approval from Ethics Committee of Zhejiang University School of Medicine, Sir Run Run Shaw Hospital (Approval NO. 20260234). The informed consent could be exempted according to the ethics committee approval letter.

### 2.2. Outcome

The incidence of recompensation was defined according to modified Baveno VII criteria: (1) Clinical resolution, defined as sustained absence of decompensation events (ascites, HE, and EGVB) for ≥12 months after discontinuation of diuretics and prophylactic therapies (e.g., rifaximin, lactulose), accompanied by restoration of Child–Pugh A liver function. Discontinuation of diuretics and prophylactic therapies was verified retrospectively through a thorough review of electronic medical records, outpatient clinic notes, and pharmacy dispensing records. (2) Aetiological suppression, demonstrated by sustained virologic response in hepatitis B/C Virus (HBV/HCV) suppression for patients with viral hepatitis, abstinence from alcohol for ≥6 months for patients with alcoholism and disease-specific control in other causes. All components required by the Baveno VII definition were systematically assessed for every patient included in the study. Data on decompensation event resolution were obtained from clinical notes and imaging records, while medication use and discontinuation were extracted from prescription and pharmacy databases. In cases where certain details were ambiguous or missing in the records, the patient was excluded to ensure strict adherence to the Baveno VII criteria. All patients were routinely followed up for 2 years after the first hospitalization for decompensated events.

During follow-up, patients who underwent liver transplantation or died before achieving recompensation were excluded from the final analysis of recompensation. This was done to ensure our analysis focused on identifying predictors of recompensation of the native liver, avoiding conflation with curative transplantation or competing risk of death.

### 2.3. Analysis of Body Composition on CT Images

**Figure 1:**
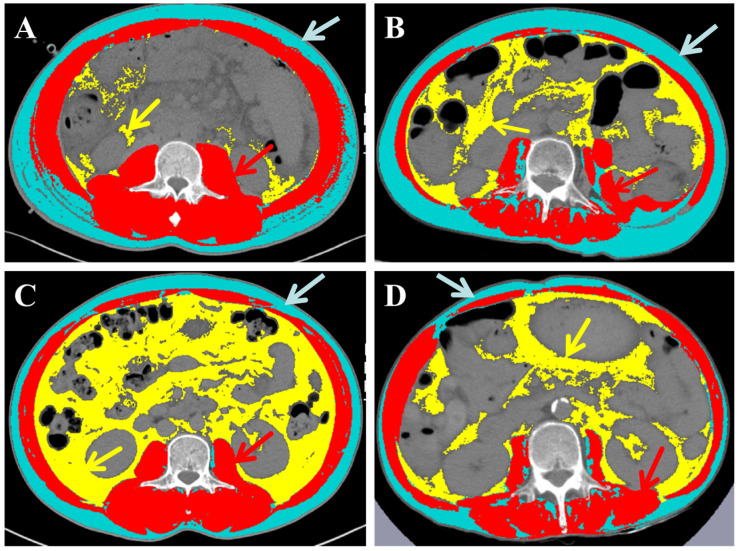
Representative CT images showing the skeletal muscle (in red), visceral adipose tissue (in yellow), and subcutaneous adipose tissue (in blue) at the L3 level for sarcopenia and visceral obesity measurements. (**A**) Normal, (**B**) isolated sarcopenia, (**C**) isolated visceral obesity, (**D**) combined sarcopenia and visceral obesity. Skeletal muscle (red arrow), visceral adipose tissue (yellow arrow), subcutaneous adipose tissue (blue arrow).

A single baseline transverse CT scan image at the third lumbar vertebra (L3) level of each patient for body composition analysis was collected from the picture archiving and communication system. To eliminate potential interobserver variability and ensure standardized measurement across the entire cohort, skeletal muscle and adipose tissue were fully automatically segmented by the ABACS module of the SliceOmatic software (v5.0, TomoVision) using predefined Hounsfield unit (HU) ranges for skeletal muscle (SM; −29 to 150 HU), visceral adipose tissue (VAT; −150 to −50 HU), and subcutaneous adipose tissue (SAT; −190 to −30 HU) (Figure 1).

Muscle area was normalized for height to derive the L3-skeletal muscle index (L3-SMI, cm^2^/m^2^). Sarcopenia was diagnosed according to validated SMI thresholds (<50 cm^2^/m^2^ for men, <39 cm^2^/m^2^ for women) [18]. The visceral-to-subcutaneous adipose tissue ratio (VSR) was calculated to indicate the relative distribution of fat (visceral versus subcutaneous propensity). Studies in various populations, including those with liver disease, indicate that VSR is a more robust predictor of adverse outcomes than VAT or SAT alone [19,20,21,22]. In line with methodological approaches used in previous research and due to the absence of a universal VSR threshold, we employed a cohort-specific, data-driven strategy [23]. Analyses were stratified by sex. Visceral obesity was defined as a VSR > 0.73 for women and >1.12 for men, the median VSR for women and men, respectively.

### 2.4. Clinical and Laboratory Assessments

Detailed demographic, clinical, and laboratory data were collected for each participant at enrollment, which included age, gender, etiology of cirrhosis, complete blood count, liver function tests, renal function tests, presence of complications and Child–Pugh classification. To account for fluid retention in cirrhotic patients, dry weight was calculated for body mass index (BMI) assessment by deducting 5%, 10%, or 15% from the measured weight for mild, moderate, or severe ascites, respectively, whereas those with peripheral edema had a uniform 5% reduction in their body weight.

### 2.5. Statistical Analyses

Between-group comparisons were performed for the overall cohort and relevant subgroups, stratified by sex, age, BMI, and Child–Pugh class. Continuous variables were compared using the Mann–Whitney U test, one-way ANOVA, or the Kruskal–Wallis test, as appropriate. Categorical variables were assessed using Fisher’s exact test or Pearson’s χ^2^ test. Factors identified as having clinically significant confounders were entered into a multivariate logistic regression model to identify independent predictors. We included 13 clinically relevant variables in the multivariable model without univariate pre-screening. To verify robustness, we performed sensitivity analyses by screening variables based on univariate *p*-values (<0.2 and <0.1). The association between combined sarcopenia, visceral obesity and non-recompensation remained consistent across all models. We therefore retained the full model as our primary analysis to minimize residual confounding. In our multivariable model, we defined non-recompensation as the binary outcome. Our model included 135 events and 13 predictors, resulting in an event-per-variable ratio of 10.4. Data are presented as median (range) unless otherwise specified, with statistical significance set at *p* < 0.05. The statistical analyses were conducted using SPSS 25.0 (SPSS Inc., Chicago, IL, USA) and GraphPad prism 9.5.1 (Graph Pad Software, La Jolla, CA, USA).

As an exploratory observational study, a formal sample size calculation was not performed a priori. All consecutive eligible patients presenting during the study period were enrolled to provide the most comprehensive data available. The study had adequate power to detect the observed significant differences in recompensation rate between key body composition subgroups.

## 3. Results

### 3.1. Patient Characteristics at Inclusion and Type of Body Composition Abnormalities

**Figure 2:**
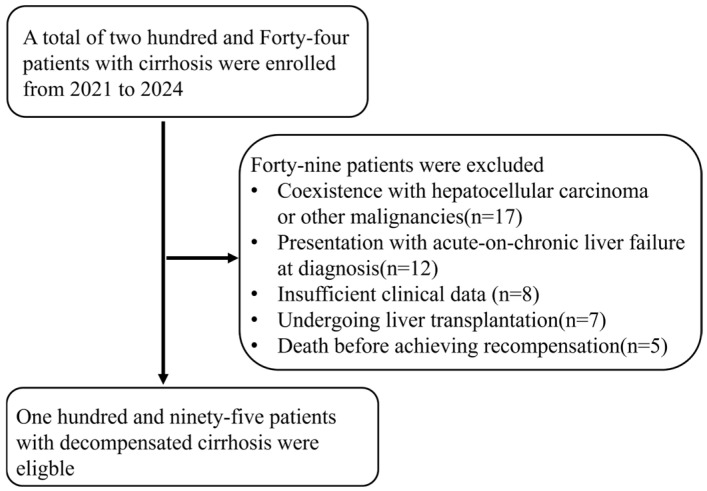
A flowchart of the study population.

A total of 244 patients with cirrhosis were enrolled in the study. Forty-nine patients were excluded because of the coexistence of hepatocellular carcinoma or other malignancies, presentation with acute-on-chronic liver failure at diagnosis, insufficient clinical data, undergoing liver transplantation, or death before achieving recompensation. Finally, we analyzed a cohort of 195 patients with decompensated cirrhosis (median age: 65 years, male: 62.05%) (Figure 2).

The predominant etiologies were chronic viral hepatitis (41.54%), alcoholism (16.92%), and autoimmune liver diseases (17.95%). Cirrhosis-related complications included ascites (69.74%), EGVB (68.72%), HE (4.62%), and infection (11.79%). Based on Child–Pugh classification, 41.03%, 52.82%, and 6.15% were categorized as A, B, and C, respectively. Body composition phenotyping revealed four distinct subgroups: normal (27.18%), I-sarco (24.62%), I-VO (26.15%), and combined sarcopenia and visceral obesity (22.05%).

During the 2-year follow-up period, 60 out of 195 patients (30.77%) achieved recompensation according to Baveno VII criteria. Table 1 presents the characteristics of patients in the total population and subgroups. Patients who achieved recompensation exhibited significant differences in age, BMI, aspartate aminotransferase (AST), albumin (ALB), ascites prevalence, Child–Pugh class, Child–Pugh score, Model for End-Stage Liver Disease (MELD) score, and phenotypic classification of body composition abnormalities distribution compared to non-recompensated patients.

### 3.2. Clinical Characteristics by Type of Body Composition Abnormality

As shown in Table 2, age showed a significant upward trend across the stratified groups, progressing from the normal group to the I-sarco/I-VO group, and peaking in the combined status group. Conversely, BMI exhibited a declining trend. In parallel, the proportion of recompensated patients decreased sequentially, reaching its lowest point in the group with both I-sarco and I-VO.

### 3.3. Factors Associated with Recompensation in Cirrhotic Patients

**Figure 3:**
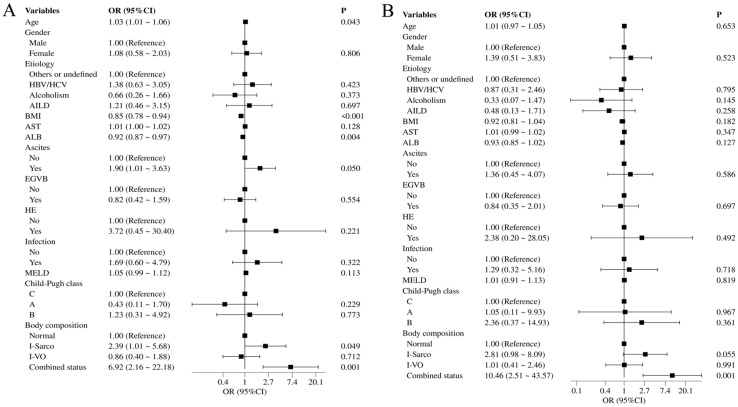
Univariate (**A**) and multivariate (**B**) logistic regression analyses to determine risk factors associated with recompensation. Abbreviations: HBV/HCV, hepatitis B/C virus; AILD, autoimmune liver diseases; BMI, body mass index; AST, serum aspartate aminotransferase; ALB, albumin; EGVB, esophagogastric variceal bleeding; HE, encephalopathy; MELD, end-stage liver disease score; I-sarco, isolated sarcopenia; I-VO, isolated visceral obesity.

In our univariate regression analysis, we identified several factors that were significantly associated with recompensation in cirrhotic patients (Figure 3A). These factors included age, BMI, ALB, and body composition abnormalities.

Subsequent multivariate analysis revealed that the status of combined sarcopenia and VO was the sole independent risk factor for non-recompensation in this population (OR 10.46, 95%CI 2.51–43.57, *p* < 0.01) (Figure 3B).

### 3.4. Subgroup Analysis of Recompensation with Body Composition Abnormalities

**Figure 4:**
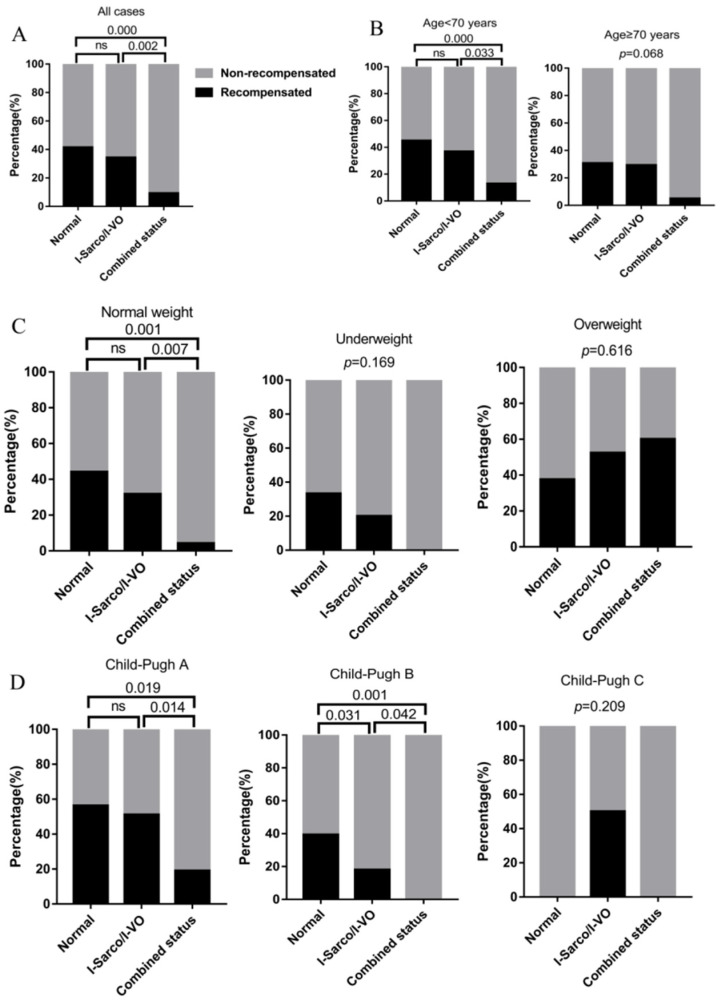
Recompensation rate stratified by body composition abnormalities in all cases (**A**) and subgroup analysis across different populations classified by age (**B**), BMI (**C**), Child–Pugh class (**D**). Abbreviations: I-sarco, isolated sarcopenia; I-VO, isolated visceral obesity. ns, not significant.

As depicted in Figure 4A, patients with combined body composition abnormalities had a significantly lower recompensation rate than those with no or isolated abnormalities (*p* < 0.05). This pattern was consistent among patients aged < 70 years, those with normal weight, and in the Child–Pugh A and B groups (*p* < 0.05) (Figure 4B–D).

## 4. Discussion

In this cohort study of hospitalized patients with decompensated cirrhosis, we observed that sarcopenia or visceral obesity, as defined by CT-quantified body compositions, were present in more than 70% of patients, and over 20% of patients had both sarcopenia and visceral obesity. Multivariate analysis revealed that sarcopenic visceral obesity was an independent risk factor for non-recompensation in patients with cirrhosis. Furthermore, the impact of sarcopenic visceral obesity on recompensation was more pronounced in subgroups of younger patients, those with normal weight, and those with preserved liver function. These findings suggest that the coexistence of these two conditions has a substantial impact on the prognosis of patients with decompensated cirrhosis, highlighting the importance of early intervention in populations with residual metabolic plasticity.

Although BMI is a convenient and commonly used metric for body composition in clinical settings, evidence regarding its association with clinical outcomes in cirrhosis remains conflicting. Some studies demonstrate that obesity is a risk factor for decompensated cirrhosis mortality [24,25,26], while others show no association [27,28,29]. This discrepancy may stem from factors that BMI fails to capture, including dynamic fluid shifts (e.g., ascites and edema) as well as sex- and race-related variations in body composition (e.g., skeletal muscle mass and fat distribution). To overcome these limitations, cross-sectional imaging is emerging as the non-invasive gold standard for body composition assessment. It enables an objective evaluation of nutritional and metabolic status through quantitative morphomics software, which utilizes standardized Hounsfield Units (HU) for precise tissue demarcation and quantification. This integrated metric of muscle and adipose tissue enhances practical risk assessment. While prior studies have employed a standard CT-quantified VAT area of ≥100 cm^2^ to define visceral obesity in cirrhotic patients with sarcopenia, this definition did not account for variations in sex or stature [30,31,32]. Furthermore, in patients with cirrhosis, the distribution of body fat may be of greater prognostic significance than overall adiposity, as evidenced by the direct correlation of VAT and the inverse correlation of SAT with clinical outcomes [22,33,34]. Thus, the CT-quantified VSR, by capturing the relative distribution of adipose tissue, may define visceral obesity more comprehensively and objectively than absolute measurements of individual fat depots or anthropometrics.

In this study, it is noteworthy that the BMI of sarcopenic visceral obesity patients was significantly lower than that of the other two groups (19.9 vs. 21.4 vs. 22.1 kg/m^2^). This further highlights the unreliability of BMI as an obesity marker. More importantly, it fails to account for weight loss due to muscle mass loss caused by sarcopenia. This loss may be masked by fluid retention in patients with ascites, which is present in most sarcopenic visceral obesity patients (65%). In this study, after adjusting for age, gender, etiology, complications, Child–Pugh class, and MELD score, sarcopenic visceral obesity remained independently associated with recompensation, whereas no such association was observed for BMI, albumin, and other factors. Therefore, identifying distinct clinical phenotypes of decompensated cirrhosis patients based on patient body composition (such as sarcopenia and visceral obesity) may help in better stratification of disease prognosis and the development of more individualized management strategies.

Due to the lack of independent predictive capacity of isolated sarcopenia, it indicates that muscle abnormality alone is insufficient to impede recompensation. Instead, visceral obesity brings about critical metabolic and inflammatory disturbances [35]. Visceral fat accumulation promotes insulin resistance, mitochondrial dysfunction, and adipokine-mediated systemic inflammation, all of which impair hepatocyte regeneration and endothelial function [36,37,38]. The liver is continuously exposed to gut-derived microbial products (e.g., lipopolysaccharide, LPS) via the portal vein. Following liver injury, resident liver cells (Kupffer cells, hepatic stellate cells, hepatocytes) upregulate toll-like receptors, especially TLR4, the main receptor for LPS. TLR activation recruits immune cells and induces pro-inflammatory cytokine production; this early inflammatory response is required to stimulate hepatocyte proliferation and activate progenitor cells for tissue repair. While low-grade TLR signaling supports regeneration, excessive or sustained activation drives persistent inflammation, hepatocyte apoptosis, and fibrosis, ultimately impairing liver regeneration and promoting dysfunction. Furthermore, the spleen may actively contribute to the pathophysiology of sarcopenia in decompensated cirrhosis through immune-mediated and metabolic pathways. Splenic congestion and immune activation in portal hypertension can amplify systemic inflammation by releasing pro-inflammatory cytokines such as TNF-α and IL-6, which promote muscle protein breakdown and inhibit synthesis [39]. Concurrently, splenic congestion exacerbates portosystemic shunting and contributes to hyperammonemia, which has been shown to upregulate myostatin—a potent negative regulator of muscle growth [40]. This inflammatory and metabolic crosstalk between the spleen and the liver may be particularly relevant in patients with sarcopenic visceral obesity and underlying NAFLD, where adipose tissue-derived inflammation and hepatic steatosis further fuel a pro-catabolic state [41]. Thus, the spleen may serve as an overlooked contributor to the “liver–muscle axis” in advanced liver disease, potentially exacerbating muscle wasting and impairing functional recovery.

The recompensation of decompensated cirrhosis is increasingly recognized as a multifactorial process involving hepatic parenchymal structural remodeling, reduction in portal hypertension, and resolution of systemic inflammation [42,43]. Although mechanistic insights are restricted by the lack of longitudinal human studies, our data provide robust clinical evidence that abnormalities in body composition quantified by CT are not only biomarkers but also modifiable determinants of liver function recovery. The translational significance of this work lies in three key contributions: (1) Defining the co-existence of sarcopenia and visceral obesity as a high-risk phenotype for recompensation; (2) Advocating for the integration of CT-quantified body composition assessment (sarcopenia and visceral obesity) into existing prognostic frameworks, potentially expanding standards such as Baveno VII to include body composition metrics; (3) Proposing multimodal interventions, including optimized protein nutrition, anti-inflammatory agents, and supervised resistance exercise, which may synergistically enhance recompensation rates in cirrhotic patients beyond standard etiology-directed and complication-focused management.

The integration of artificial intelligence (AI) and automated image processing has transformed the clinical management of liver cancer and cirrhosis, overcoming longstanding limitations of conventional manual assessment. In diagnosis, AI enables accurate, reproducible, automated quantification of cirrhosis features and early detection of hepatocellular carcinoma (HCC) from CT/MRI images, outperforming visual assessment in identifying small or early-stage lesions and reducing observer variability. For treatment planning, AI improves the precision of surgical and interventional procedures by automatically delineating tumors and critical structures, predicts procedural risk for cirrhotic patients, and enables earlier monitoring of treatment response. In prognostication, AI combines radiomic image features with clinical data to deliver more accurate risk stratification than traditional staging systems, predicting decompensation, HCC development, and tumor recurrence to guide personalized surveillance and treatment. While major challenges (including multi-center validation, standardization, and regulatory approval) remain, AI and image processing are set to become core tools for personalized care, improving objectivity, efficiency and patient outcomes across the full management pathway for liver diseases.

Several limitations are noted. Primarily, the retrospective nature and single-center setting of this study potentially affect patient selection and may restrict the external validity of the results. Second, the sample sizes of some subgroups are relatively small, particularly for patients with Child–Pugh C cirrhosis and those with the combined phenotype of sarcopenia and visceral obesity. This distribution reflects the clinical epidemiology of our cohort. The small size of the subgroups may limit the statistical power to detect subgroup-specific effects. Third, body composition assessment relied on CT-derived quantitative parameters without concurrent evaluation of muscle function (e.g., grip strength, gait speed, or chair stand time). Finally, key potential confounding factors were not systematically collected or adjusted for in the regression models, including concomitant medications (e.g., beta-blockers, diuretics, or anti-inflammatory drugs), detailed nutritional intake data (e.g., protein and energy expenditure), and patient-reported outcomes (e.g., fatigue or physical function). Prospective, multi-center studies incorporating standardized functional body composition assessments and mechanistic biomarkers are needed to validate these findings and elucidate causal pathways. Nevertheless, our results indicate a significant association between abnormal body composition (coexistence of sarcopenia and visceral obesity) and recompensation in patients with decompensated cirrhosis.

## 5. Conclusions

Our findings demonstrate that sarcopenic visceral obesity is a significant and independent risk factor for non-recompensation in decompensated cirrhosis. Clinicians should be aware that patients may present with this combined condition, necessitating a targeted approach concerning their management. The prospect of improving body composition abnormalities presents a tempting strategy with clinical implications.

## Figures and Tables

**Table 1:** Baseline characteristics of enrolled patients with decompensated cirrhosis.

Variables	Total (*n* = 195)	Recompensated (*n* = 60)	Non-Recompensated (*n* = 135)	*p*
Age (years)	65 (58, 72)	62 (55.75, 69.5)	67 (59, 72.5)	0.039
Gender, *n* (%)				0.806
male	121 (62.05)	38 (63.33)	83 (61.48)	
female	74 (37.95)	22 (36.67)	52 (38.52)	
Etiology, *n* (%)				0.369
HBV/HCV	81 (41.54)	21 (35.00)	60 (44.44)	
Alcoholism	33 (16.92)	14 (23.33)	19 (14.07)	
AILD	35 (17.95)	10 (16.67)	25 (18.52)	
Others or undefined	46 (23.59)	15 (25.00)	31 (22.96)	
BMI	21.4 (19.4, 23.65)	22.8 (19.9, 25.13)	21 (19.2, 23.1)	0.002
WBC (×10^9^/L)	4.1 (2.6, 6.2)	3.70(2.55, 5.5)	4.50(2.65, 6.65)	0.132
HG (g/L)	98(80, 120)	98.5 (84.75, 116.75)	98 (78, 120)	0.266
PTL (×10^9^/L)	77 (53, 114)	73.5 (56, 98.5)	79 (52.5, 116)	0.698
ALT (U/L)	25 (18, 37)	22.5 (18, 29.25)	25 (18, 40)	0.257
AST (U/L)	37 (28.5, 53)	32 (27.75, 40.25)	39 (30, 58)	0.004
ALB (g/L)	33 (30.15, 37.2)	35.2 (32.6, 38.75)	32.3 (29.25, 35.95)	0.002
TBIL (µmol/L)	21.7 (14.8, 34.15)	20.45 (15.78, 32.6)	21.7 (14.1, 35.95)	0.921
Crea (µmol/L)	70 (58, 81)	73 (59.5, 82.25)	67 (57, 80.5)	0.277
Complications, *n* (%)				
Ascites	136 (69.74)	36 (60)	100 (74.07)	0.048
EGVB	134 (68.72)	43 (71.67)	91 (67.41)	0.554
HE	9 (4.62)	1 (1.67)	8 (5.93)	0.348
Infection	23 (11.79)	5 (8.33)	18 (13.33)	0.318
Child–Pugh class, *n* (%)			0.005	
A	80 (41.03)	35 (58.33)	45 (33.33)	
B	103 (52.82)	22 (36.67)	81 (60.00)	
C	12 (6.15)	3 (5.00)	9 (6.67)	
Child–Pugh score	7(6, 8)	6 (5, 8)	7 (6, 8)	0.009
MELD	9 (7.5, 12)	9 (5.75, 11)	10 (8, 12)	0.041
Body composition, *n* (%)				<0.001
Normal	53 (27.18)	22 (36.67)	31 (22.96)	
I-Sarco	48 (24.62)	11 (18.33)	37 (27.41)	
I-VO	51 (26.15)	23 (38.33)	28 (20.74)	
Combined status	43 (22.05)	4 (6.67)	39 (28.89)	

Abbreviations: HBV/HCV, hepatitis B/C virus; AILD, autoimmune liver diseases; BMI, body mass index; WBC, white blood cell count; HG, hemoglobin; PLT, platelet; ALT, serum alanine aminotransferase; AST, serum aspartate aminotransferase; ALB, albumin; TBIL, total serum bilirubin; Crea, creatinine; EGVB, esophagogastric variceal bleeding; HE, encephalopathy; MELD, end-stage liver disease score; I-sarco, isolated sarcopenia; I-VO, isolated visceral obesity.

**Table 2:** Clinical characteristics between phenotypic classification of body composition.

Variables	Normal (*n* = 53)	I-Sarco/I-VO (*n* = 99)	Combined Status (*n* = 43)	*p*
Age	61 (56, 69)	66 (57, 71.5)	68 (63.5, 73)	0.005
Gender, *n* (%)				0.545
male	30 (56.60)	62 (62.63)	29 (67.44)	
female	23 (43.40)	37 (37.37)	14 (32.56)	
Etiology, *n* (%)				0.084
HBV/HCV	22 (41.51)	35 (35.35)	24 (55.81)	
Alcoholism	9 (16.98)	18 (18.18)	6 (13.95)	
AILD	7 (13.21)	18 (18.18)	10 (23.26)	
Others or undefined	15 (28.30)	28 (28.28)	3 (6.98)	
BMI	22.1 (20.6, 25.3)	21.4 (19.5, 23.65)	19.9 (17.5, 22.1)	<0.001
AST	38 (28, 57)	35 (28.5, 46)	39 (29.5, 56)	0.540
ALB	32.9 (29.1, 36.2)	32.9 (30.45, 36.95)	33.4 (29.55, 37.55)	0.554
Complications, *n* (%)				
Ascites	39 (73.58)	69 (69.70)	28 (65.12)	0.668
EGVB	36 (67.92)	66 (66.67)	32 (74.42)	0.651
HE	1 (1.89)	6 (6.06)	2 (4.65)	0.602
Infection	5 (9.43)	11 (11.11)	7 (16.28)	0.560
Child–Pugh class, *n* (%)				0.401
A	16 (30.19)	43 (43.43)	21 (48.84)	
B	33 (62.26)	50 (50.51)	20 (46.51)	
C	4 (7.55)	6 (6.06)	2 (4.65)	
Child–Pugh score	7 (6, 8)	7 (6, 8)	7 (5, 8)	0.056
MELD	10 (8, 13)	10 (7.5, 12)	9(7, 10.5)	0.191
Recompensation, *n* (%)	22 (41.51)	34 (34.34)	4 (9.30)	0.002

Abbreviations: HBV/HCV, hepatitis B/C virus; AILD, autoimmune liver diseases; BMI, body mass index; AST, serum aspartate aminotransferase; ALB, albumin; EGVB, esophagogastric variceal bleeding; HE, encephalopathy; MELD, end-stage liver disease score; I-sarco, isolated sarcopenia; I-VO, isolated visceral obesity.

## Data Availability

The original contributions presented in this study are included in the article. Further inquiries can be directed to the corresponding author.
